# Imaging features of intraductal tubulopapillary neoplasm of the pancreas and its differentiation from conventional pancreatic ductal adenocarcinoma

**DOI:** 10.1038/s41598-022-19517-6

**Published:** 2022-09-16

**Authors:** Ekaterina Khristenko, Thomas Hank, Matthias M. Gaida, Hans-Ulrich Kauczor, Thilo Hackert, Miriam Klauß, Philipp Mayer

**Affiliations:** 1 Clinic for Diagnostic and Interventional Radiology, Heidelberg University Hospital, Im Neuenheimer Feld 420, 69120 Heidelberg, Germany; 2 Division of Visceral Surgery, Department of General Surgery, Medical University of Vienna, Vienna, Austria; 3 Institute of Pathology, University Medical Center Mainz, JGU-Mainz, 55131 Mainz, Germany; 4 Department of General, Visceral, and Transplantation Surgery, Heidelberg University Hospital, 69120 Heidelberg, Germany

**Keywords:** Cancer, Medical research, Oncology

## Abstract

Intraductal tubulopapillary neoplasms (ITPN) are rare pancreatic tumors (< 1% of exocrine neoplasms) and are considered to have better prognosis than classical pancreatic ductal adenocarcinoma (PDAC). The present study aimed to evaluate imaging features of ITPN in computed tomography (CT) and magnetic resonance (MR) imaging. We performed monocentric retrospective analysis of 14 patients with histopathologically verified ITPN, operated in 2003–2018. Images were available for 12 patients and were analysed independently by two radiologists, blinded to reports. Imaging features were compared to a matched control group consisting of 43 patients with PDAC, matched for sex and age. Histopathologic analysis showed invasive carcinoma component in all ITPN patients. CT-attenuation values of ITPN were higher in arterial and venous phases (62.3 ± 14.6 HU and 68 ± 15.6 HU) than in unenhanced phase (39.2 ± 7.9 HU), compatible with solid lesion enhancement. Compared to PDAC, ITPN lesions had significantly higher HU-values in both arterial and venous phases (arterial and venous phases, p < 0.001). ITPN were significantly larger than PDAC (4.1 ± 2.0 cm versus 2.6 ± 0.84 cm, p = 0.021). ITPN lesions were more often well-circumscribed (p < 0.002). Employing a multiple logistic regression analysis with forward stepwise method, higher HU density in the arterial phase (p = 0.012) and well-circumscribed lesion margins (p = 0.047) were found to be significant predictors of ITPN versus PDAC. Our study identified key imaging features for differentiation of ITPN and PDAC. Isodensity or moderate hypodensity and well-circumscribed margins favor the diagnosis of ITPN over PDAC. Being familiar with CT-features of these rare pancreatic tumors is essential for radiologists to accelerate the diagnosis and narrow the differentials.

## Introduction

Intraductal tubulopapillary neoplasms (ITPN) are rear tumors of the pancreas, accounting for less than 1% of all exocrine neoplasms, which were included in 2010 to the 4th edition of the World Health Organization (WHO) Classification^1^. They are characterized predominantly by an intraductal growth pattern, with only minimal papilla formation. Instead, the neoplastic epithelial cells are filling the ducts with back-to-back tubular glands, without extensive luminal or intracellular mucin accumulation^2^.

In the case of R0 resection, ITPN are considered to have a better prognosis than pancreatic ductal adenocarcinoma (PDAC), even if an associated carcinoma is detected^3,4^. In case of an associated invasive carcinoma, the 5-year survival rate is greater than 70%^2^.

The current WHO classification recognizes four categories of primary intraductal neoplasms of the pancreas: pancreatic intraepithelial neoplasia (PanIN), intraductal papillary mucinous neoplasms (IPMN), intraductal oncocytic papillary neoplasm (IOPN), and ITPN. IOPN, are the oncocytic subtype of IPMN, but due to their different biological behavior, they are considered as own entity. PanIN are microscopic lesions that usually cannot be visualized on radiologic imaging. By contrast, ITPN, IPMN, IOPN appear as focal pancreatic lesions in CT and MRI^5^.

The clinical, pathological, and radiological characteristics of IPMN have been investigated extensively during the past years. With recent improvements in imaging techniques, pancreatic intraductal lesions, especially IPMN, are being detected with increasing frequency^6–10^. By contrast, there is limited knowledge on ITPN as precancerous lesions and on the invasive carcinoma arising from ITPN. Due to the rarity of these lesions, data on the natural history of ITPN and its clinical and radiological features are available mainly as case reports with review of the literature^4,11–16^. Thus, ITPN remains a challenging diagnosis for both pathologists and radiologists. Previous reports indicated that, on contrast-enhanced CT, ITPNs with associated invasive carcinoma can appear as ill-defined hypodense focal pancreatic lesions^17,18^. This appearance is similar to that of conventional PDAC^19^ and some authors reported preoperative misdiagnosis of ITPN with associated invasive carcinoma as conventional PDAC^17,20^. Various previous studies have focused on imaging features for differentiation of other pancreatic lesions from conventional PDAC: The “duct-penetrating sign” has a high accuracy for the diagnosis of mass-forming chronic pancreatitis^21^. Solid pseudopapillary neoplasms commonly present as large tumors with hemorrhagic degeneration^22^. Most pancreatic neuroendocrine neoplasms are well-defined and typically iso- or hyperdense after contrast injection due to their vascularization^23^. The absence of ductal dilatation and peripancreatic infiltration are helpful findings to distinguish non-hypervascular PNETs from conventional PDAC^24^. However, studies on imaging differentiation of ITPN with associated invasive carcinoma from conventional PDAC are lacking.

The aim of the study is to evaluate imaging features of pancreatic intraductal tubulopapillary neoplasms in CT and MRI, in comparison to PDAC.

## Results

Sex, age, and lesion distribution, as well as other characteristics of the patients with ITPN are presented in Table 1. The mean age in the ITPN-group was 63.8 ± 13.1 years (mean age ± standard deviation), the median age was 64 years (interquartile range (IQR) 56–73 years). The mean age in the group of PDAC was 64.1 ± 8.6 years, the median age was 64 years (IQR 58–71 years). All ITPN patients underwent surgical resection of the tumors: ten patients underwent pancreatoduodenectomy and two patients underwent left resection. Histopathologic analysis showed an invasive carcinoma component in all patients; pathological lesion size ranged from 1.5 to 4.0 cm.

**Table 1:** Sex, age, imaging characteristics, and suspected preoperative diagnosis of the ITPN-patients.

Case	Age/sex	Pathology	Available imaging	Location	Lesion size (cm)	Diameter MPD (mm)	Attenuation characteristics (venous phase)	Suspected preoperative diagnosis
1	36/m	ITPN with associated carcinoma (AC)	CT and MRI	Head	5.5	12	Isodense/-intense	Solid lesion
2	61/m	ITPN with AC	MRI	Head	6.3	10	NA	PDAC
3	87/f	ITPN with AC	CT	Head	8.7	4	Hypodense	MCN
4	63/m	ITPN with AC	CT	Head	5.4	5	Isodense	Solid lesion
5	65/m	ITPN with AC	CT and MRI	Head	2.1	4	Hypodense/-intense	PDAC
6	72/f	ITPN with AC	CT and MRI	Head	2.3	9	Hypodense/-intense	Solid lesion with ductal component
7	74/f	ITPN with AC	CT	Head	3	6	Isodense	Isoattenuating PDAC
8	58/f	ITPN with AC	CT and MRI	Tail	2.3	3	Hypodense/-intense	Intrapancreatic cystic lesion
9	68/m	ITPN with AC	CT	Head	2.8	4	Isodense	Isoattenuating PDAC
10	52/m	ITPN with AC	CT and MRI	Head	4.1	10	Hypodense/-intense	PDAC
11	75/f	ITPN with AC	MRI	Body	3.3	10	NA	MT-IPMN with ductal component
12	54/m	ITPN with AC	CT and MRI	Head	3.1	12	Isodense/-intense	Solid lesion with ductal component

*MPD* main pancreatic duct, *ITPN* intraductal tubulopapillary neoplasm, *CT* computed tomography, *MRI* magnetic resonance imaging, *NA* not available, *PDAC* pancreatic ductal adenocarcinoma, *MCN* mucinous cystic neoplasm.

### Imaging findings

Ten out of twelve ITPN lesions were located in the pancreatic head, one ITPN lesion in the pancreatic body, and one in the pancreatic tail. In comparison to PDAC lesions (28 out of 43 in the pancreatic head, 7 out of 43 in the pancreatic body, and 8 out of 43 in the pancreatic tail), there was no difference found between the groups (p = 0.232).

The mean size of the ITPN lesions on imaging was 4.1 ± 2.0 cm (size range 2.1 to 8.7 cm). The mean size PDAC lesions on imaging was 2.6 ± 0.84 cm (size range 1.1 to 4.4 cm). We found a statistically significant difference between the groups, when compared by lesion size (p = 0.021).

**Figure 1:**
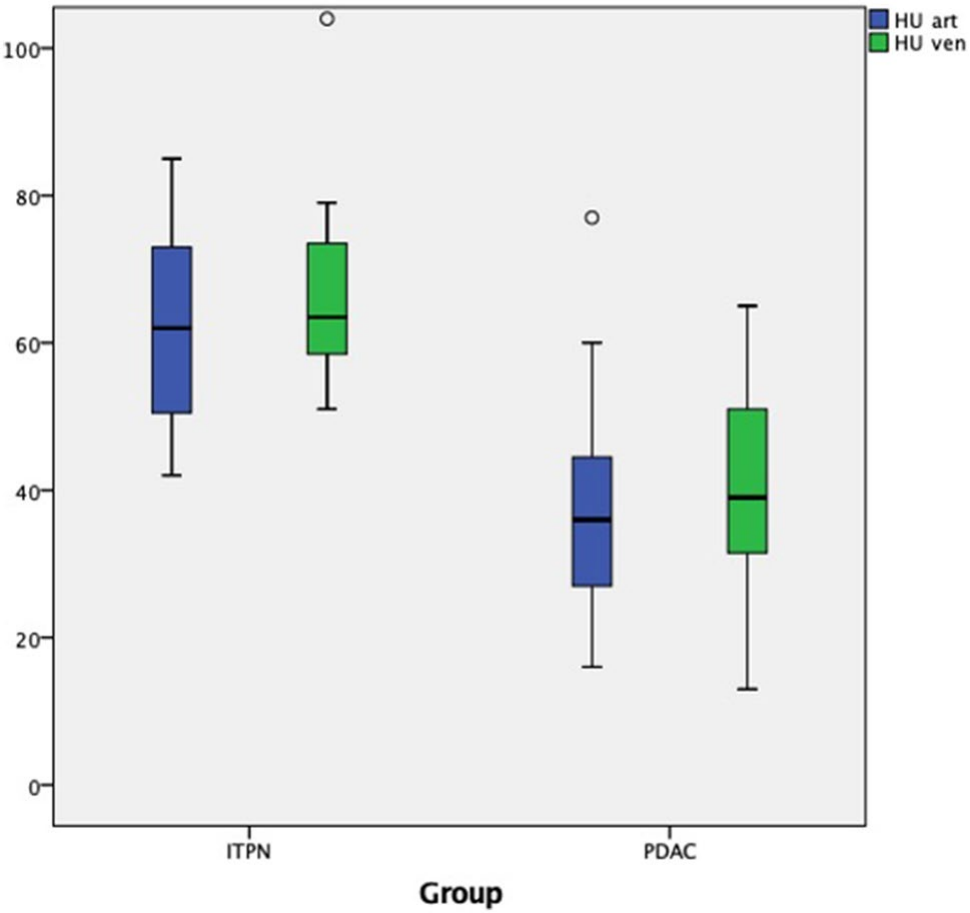
Box plots demonstrating attenuation differences in the group with ITPN and PDAC in the arterial and venous phases.

Mean HU values of ITPN lesions in the unenhanced phase were 39.2 ± 7.9 HU, in the arterial phase 62.3 ± 14.6 HU, and in the venous phase 68.0 ± 15.6 HU. Thus, the cystic morphology of the ITPN lesions was not verified using computed tomography. In comparison to PDAC, ITPN lesions had significantly higher HU values in both arterial and venous phases (arterial phase: mean HU values ITPN 62.3 ± 14.6 HU, mean HU values PDAC 34.4 ± 12.3 HU with p < 0.001; venous phase: mean HU values ITPN 68 ± 15.6 HU, mean HU values PDAC 40.1 ± 13.6 HU with p < 0.001), which is demonstrated in Fig. 1. PDAC were relatively hypodense in the arterial and venous phase compared to the non-neoplastic pancreatic parenchyma with mean HU values of the non-neoplastic pancreatic parenchyma 93.5 ± 29.7 HU and 87.4 ± 19.6 HU, respectively.

The dilatation of the main pancreatic duct was defined as dilatation ≥ 5 mm and was highly prevalent in both groups, accounting for 66.7% of ITPN patients (8 out of 12 patients) and 76.7% of PDAC patients (33 out of 43 patients). 37.5% of these 8 ITPN were associated with a moderate upstream dilatation of the main pancreatic duct (diameter 5 to 9 mm) and 62.5% showed a marked upstream dilatation of the main pancreatic duct (≥ 10 mm). In the group of PDAC patients with ductal dilatation, the clear majority of lesions (75.8%) were associated with a moderate upstream pancreatic duct dilatation while 24.2% of lesions showed a marked upstream duct dilatation. The mean duct diameter in the ITPN group was 7.4 ± 3.4 mm and in the group of PDAC 7.1 ± 3.9 mm. The difference was not statistically significant according to the Mann–Whitney U-test (p = 0.645). The relative frequencies of no duct dilatation (< 5 mm), moderate duct dilatation (5 to 9 mm), and marked duct dilatation (≥ 10 mm) were not statistically different between the ITPN group and PDAC group (Chi-squared test, p = 0.105).

We then performed a subgroup analysis including only lesions located in the pancreatic head. Out of 10 patients with ITPN lesions in the pancreatic head, three (30.0%) showed a moderate and four (40.0%) a marked upstream dilatation of the main pancreatic duct. Out of 28 patients with PDAC lesions in the pancreatic head, a moderate duct dilatation was observed in twenty patients (71.4%) and a marked duct dilatation in three patients (10.7%). In this subgroup analysis, the relative frequencies of no duct dilatation, moderate and marked duct dilatation were significantly different between the ITPN group and PDAC group (p = 0.049, chi-squared test). However, the difference in duct diameters was not statistically significant according to the Mann–Whitney U-test (7.6 ± 3.3 mm for ITPN, 7.3 ± 3.9 mm for PDAC, Mann–Whitney U-test, 0.605). Eight out of ten (80.0%) ITPN lesions in the pancreatic head and 23 out of 28 (82.1%) PDAC lesions in the pancreatic head were associated with an abrupt change in duct diameter (p = 0.882, chi-squared test).

**Figure 2:**
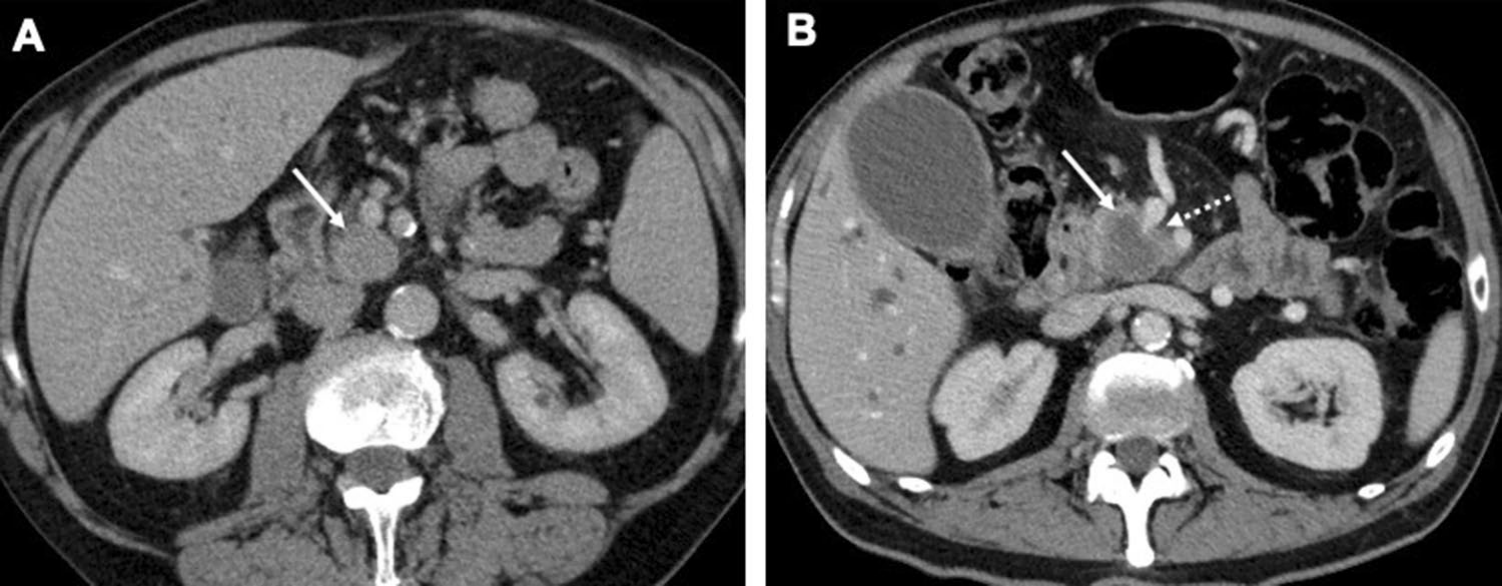
Comparison of CT features of ITPN with associated invasive carcinoma and PDAC. (**A**) Axial CT scan in the portal venous phase showing ITPN lesion in the pancreatic head (arrow) with well-circumscribed margins and isodense HU values. Note the preserved morphology and boundaries of the superior mesenteric vessels. (**B**) Axial CT scan of a different patient in the portal venous phase showing PDAC in the same location (arrow) showing unsharp margins, hypodensity und classical tear-drop sign of superior mesenteric vein (dotted arrow), suggesting its infiltration.

ITPN lesions were more often well-circumscribed, compared to PDAC lesions (p < 0.002). Figure 2 shows a comparison of CT morphology of ITPN and PDAC.

**Figure 3:**
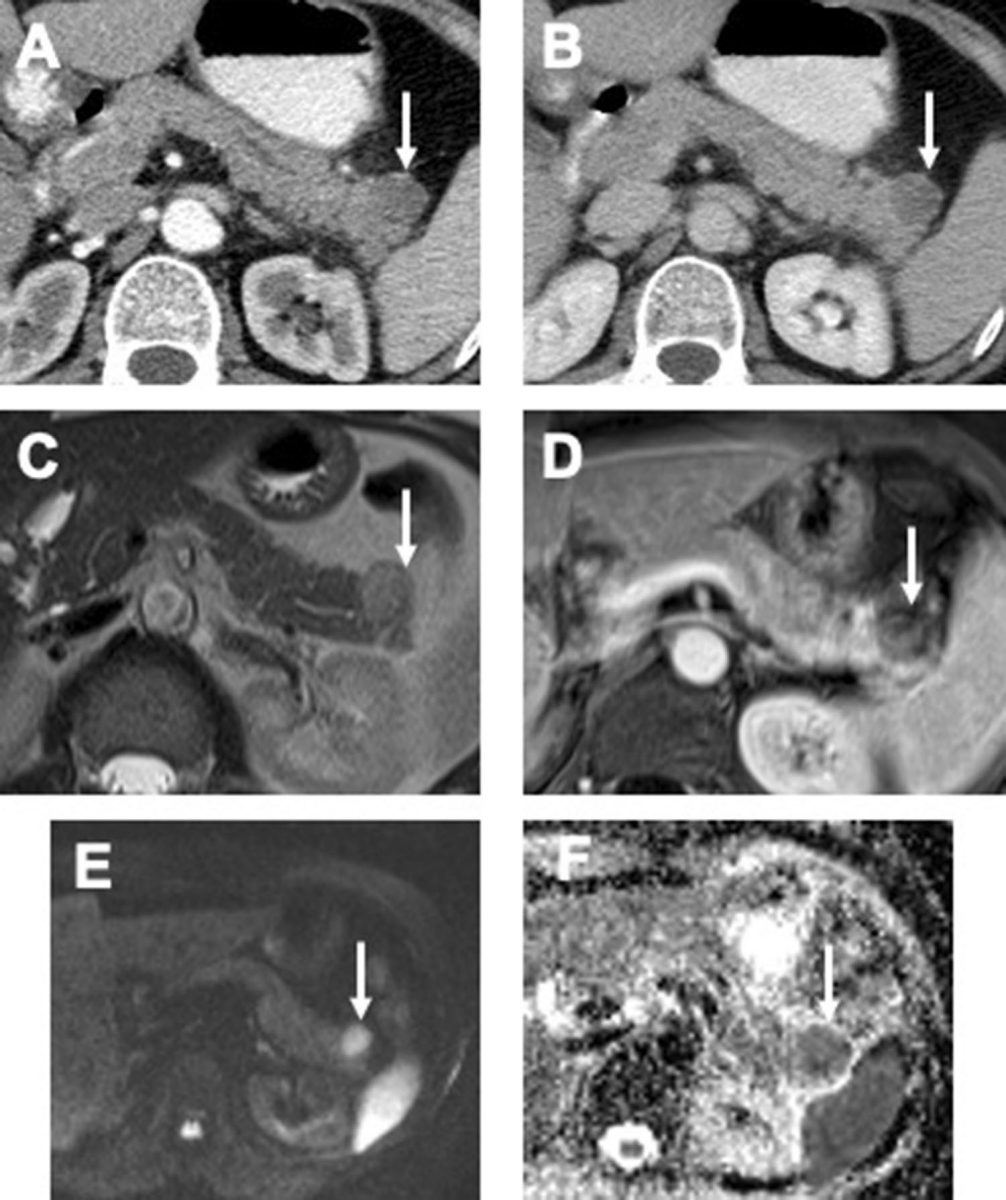
CT and MRI features of ITPN with associated invasive carcinoma. (**A,B**) Axial CT scans in the arterial phase (**A**) and portal venous phase (**B**) demonstrating ITPN lesion in the pancreatic tail (arrows) with well-circumscribed margins and moderate hypointense HU values. (**C**) Axial T2 weighted image of the same patient, tumor (arrow) doesn’t have classical cystic morphology on T2 but is slightly T2 hyperintense compared to pancreatic parenchyma. (**D**) Axial T1 weighted image with fat suppression after CM administration, portal venous phase. Tumor (arrow) shows low inhomogeneous contrast enhancement without invasion of adjacent structures. (**E,F**) Tumor (arrows) shows hyperintensity on axial DWI with b-value 800 s/mm^2^ (**E**) and hypointensity on ADC map (**F**), which corresponds the restricted diffusion.

In all 8 ITPN cases with available MRI examinations, the lesions were hypointense on non-contrast T1WI and were iso- to slightly hyperintense on T2WI. DWI was available for 6 ITPN lesions and all of them showed a restricted diffusion (Fig. 3).

**Figure 4:**
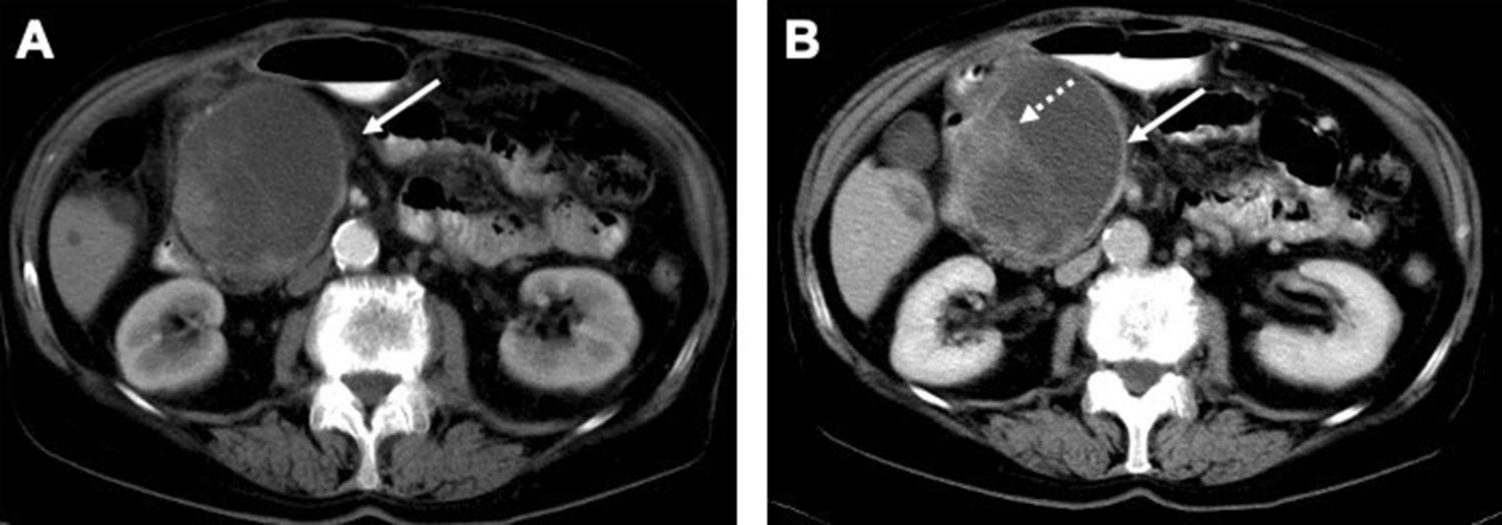
ITPN mimicking the morphology of mucinous cystic neoplasm. (**A,B**) Axial CT scans in the arterial phase (**A**) and portal venous phase (**B**) demonstration a huge oligocystic mass (arrow) in the pancreatic head with an enhancing solid nodular component (dotted arrow).

In 10 ITPN cases, the lesions didn’t show cystic signal intensities in MRI or density values in CT. Two ITPN lesions exhibited cystic morphology on radiological imaging. Out of the two cases with cystic imaging morphology, the first lesion presented as an oligocystic mass with an enhancing solid nodular component. In this case, a suggested preoperative diagnosis was mucinous cystic neoplasm (MCN) or huge branch duct IPMN (Fig. 4).

**Figure 5:**
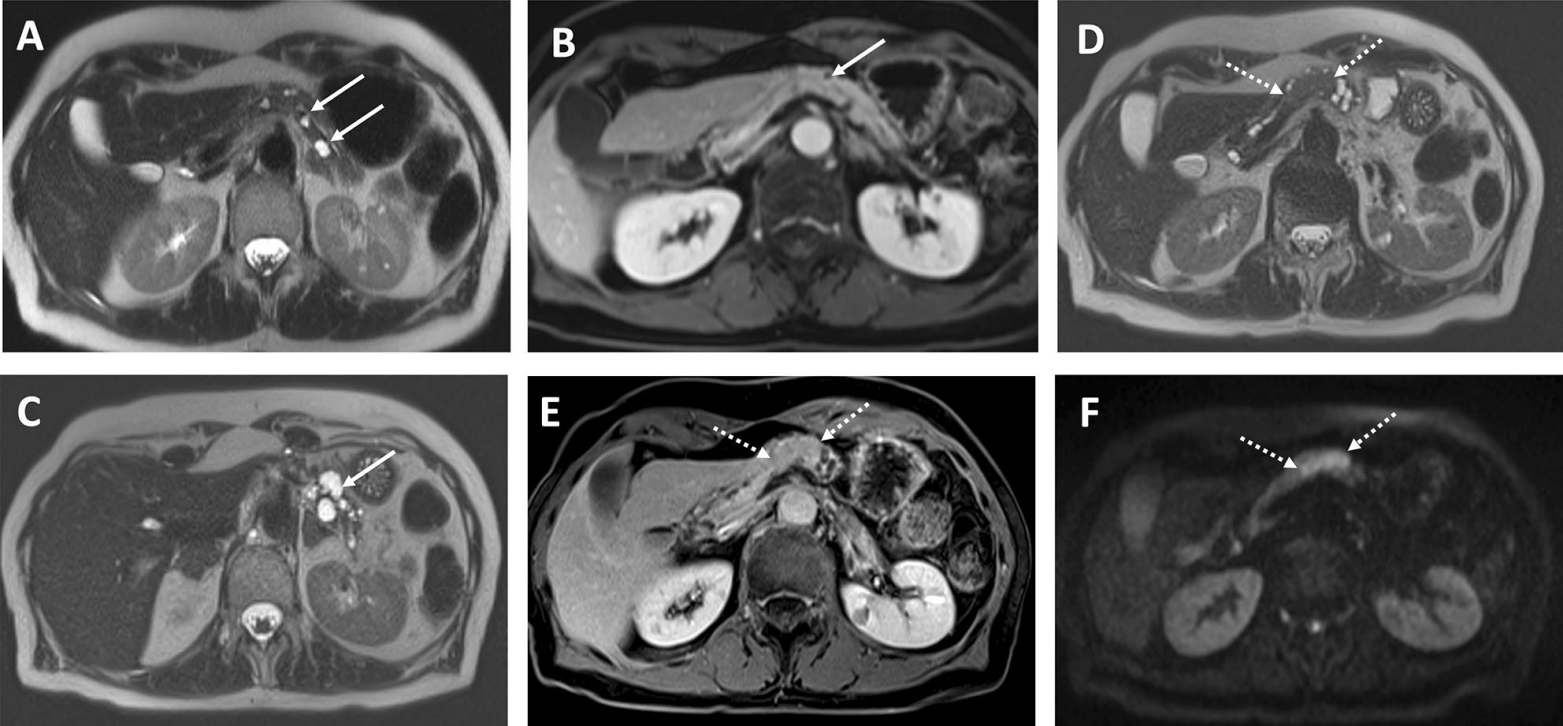
Cystic pancreatic lesions with development of solid ITPN in the pancreatic body. (**A,B**) Initial MRI study with cystic pancreatic lesions. (**A**) Axial T2 weighted image shows small cystic lesions (arrows) in the pancreatic tail without dilatation of the main pancreatic duct, suggesting small branch duct IPMNs. (**B**) Axial T1 weighted image after contrast medium administration in the portal venous phase. Tiny cystic lesion without contrast enhancement (arrow). No evidence of the tumor in the pancreatic body. (**C–F**) Follow-up of the same patient in 7 years. (**C**) Axial T2 weighted image, known cystic lesions in the pancreatic tail significantly increased in size (arrow). (**D**) Axial T1 weighted image after contrast medium administration in the portal venous phase. New lesion in the pancreatic body showing solid pattern of contrast enhancement being almost isointense to pancreatic parenchyma. (**E**) Axial T2 weighted image, elongated solid lesion within the pancreatic body (dotted arrows), which was histologically confirmed as ITPN after resection. (**F**) DWI with b-value 800 s/mm^2^ with restricted diffusion within the lesion.

The second lesion exhibited an IPMN-like cystic component in the pancreatic tail and a solid component in the pancreatic body. This patient had been followed-up by imaging for 7 years before surgery. The initial MRI scans had shown small cystic lesions in the pancreatic tail without dilatation of the main pancreatic duct, suggesting small branch duct IPMNs. Within the 7 years follow-up period, a solid intraductal component developed in the pancreatic body with upstream dilatation of the main and branch pancreatic ducts, mimicking a mixed-type IPMN (Fig. 5).

Employing a multiple logistic regression analysis with forward stepwise method (n = 39 patients), higher HU density in the arterial phase (p = 0.012) and sharp lesion margins (p = 0.047) were found to be significant predictors of ITPN lesions versus conventional PDAC lesions. Other independent variables were not included in the final model: HU density in the venous phase, size of the lesion, and location of the lesion.

**Figure 6:**
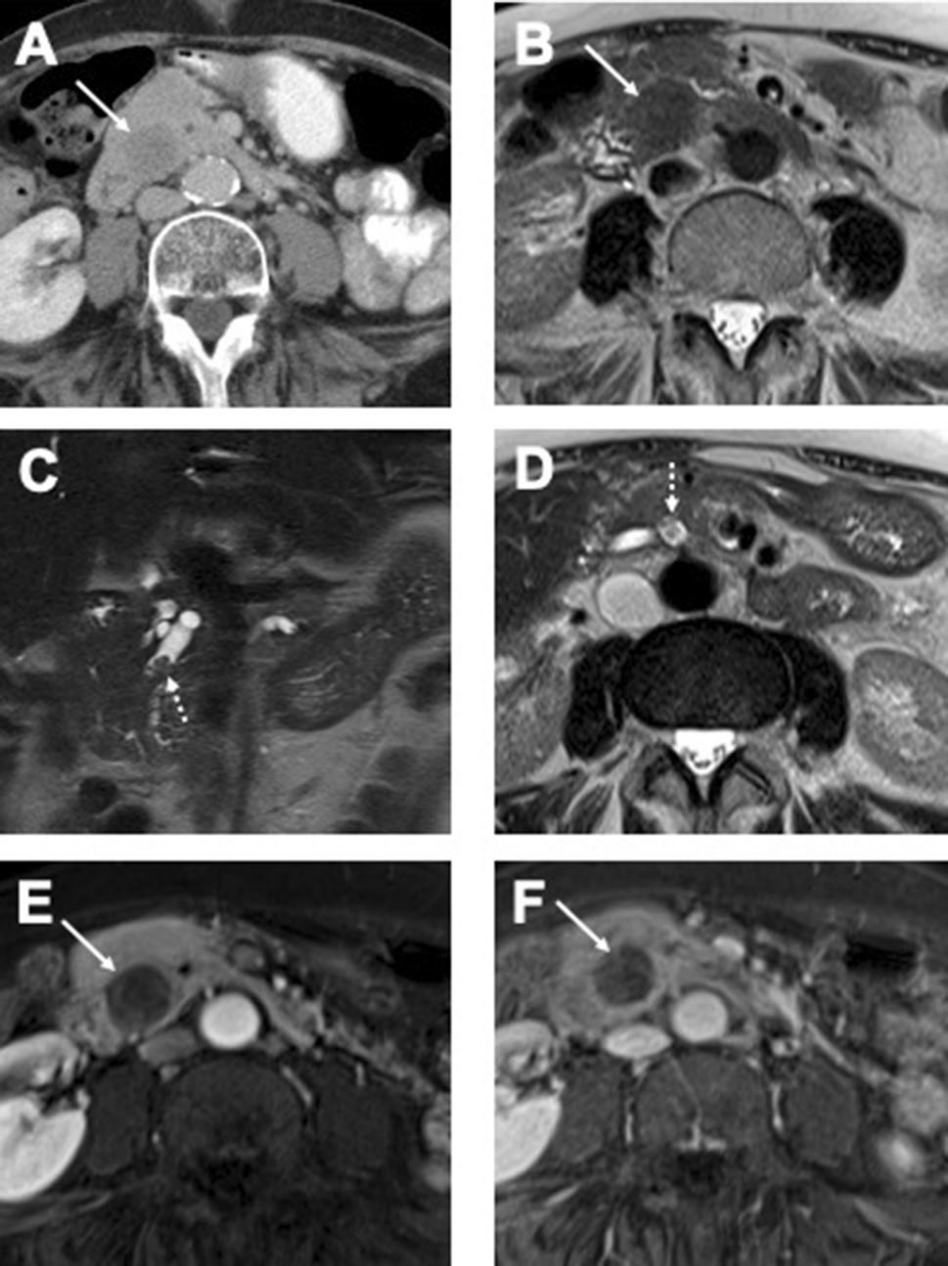
CT and MRI features of ITPN with intraductal solid tumor growth. (**A**) Axial CT scan in the portal venous phase shows an ITPN lesion in the pancreatic head (arrow) with well-circumscribed margins and slightly hypodense HU-values, compared to normal pancreatic parenchyma. (**B**) Axial T2 weighted image of the same patient. The lesion had T2 hypointense signal (arrow), suggesting a solid morphology. (**C,D**) Coronal T2 weighted image (**C**) and axial T2 weighted image (**D**) show dilated main pancreatic duct with intraductal solid tumor growth (dotted arrows). (**E,F**) T1 weighted images with fat suppression after contrast administration in the arterial (**E**) and venous (**F**) phase. In both phases tumor is hypointense to pancreatic parenchyma without significant contrast enhancement.

Intraductal solid tumor growth (previously reported as 2-tone duct sign) was seen in three patients. In one of them, it was present simultaneously with a parenchymatous solid pancreatic lesion, which is shown in Fig. 6.

One out of twelve ITPN lesions was morphologically indistinguishable from classic PDAC by both CT and MR examinations, appearing as a hypovascular solid lesion within the pancreatic head, marked upstream duct dilatation, and distal atrophy of the parenchyma. Locoregional lymphadenopathy and focal liver lesions of uncertain etiology were also present. Histologically ITPN-associated low differentiated adenocarcinoma of the pancreas was found. On histopathology, there was an infiltration of the peripancreatic fat, the duodenum, and the partially resected portal vein.

### Progression-free survival

Follow-up data regarding progression-free survival were available for four patients with ITPN with associated invasive carcinoma and 24 patients with conventional PDAC. Out of the four patients with ITPN with associated invasive carcinoma, progression occurred 130 days (distant metastases) and 1478 days (local recurrence) after surgery; the remaining two patients had no signs of disease progression after a follow-up of 581 days and 2457 days. Out of the twenty-four patients with conventional PDAC, progression was detected in seventeen patients with a median progression-free survival of 292 days (95% confidence interval: 149 to 618 days) after surgery (local recurrence in seven patients, distant metastases in seven patients, both in three patients); in the remaining seven patients, follow-up CT-scans showed no evidence of progression after a median follow-up of 610 days (range 196 to 1842 days). Progression-free survival was longer in patients with ITPN with associated carcinoma than in patients with conventional PDAC (p = 0.1969, log-rank test). Kaplan–Meier curves are presented in Supplementary Fig. 1. The survival data presented here should be regarded as descriptive since ITPN and PDAC cases were not matched according to tumor stage.

## Discussion

Intraductal tubulopapillary neoplasms of the pancreas are rare intraductal pancreatic tumors with limited data on their natural course as well as their clinical, histopathological, and radiological features. Compared to PDAC, they were reported to have a better prognosis, with a 5-year survival rate greater than 70%, even with the presence of an invasive component^2,4^. This is markedly higher than the 9% 5-year survival rate of PDAC^25,26^. According to previous reports, ITPN showed an invasive component in up to 50–60% of cases on histopathology^12,27,28^. Rooney et al.^27^ found an invasive component in 13 out of 24 patients (54%). By contrast, in our study, all ITPN exhibited an associated invasive carcinoma component. This discrepancy could possibly be explained by referral bias. Patients in tertiary referral centers, like our hospital, often present with more advanced stages of disease^29^.

Motosugi et al.^30^ described radiological features of 11 ITPN, of which upstream dilatation of the main pancreatic duct was present in 10 patients (one patient with a branch duct lesion had no dilatation). The authors suggested that the absence of downstream dilatation of the main pancreatic duct in ITPN is a key imaging finding for differentiation from IPMN/IOPN which exhibit downstream dilation due to abundant mucin production. In our patient cohort, marked upstream dilatation was present in 50% of ITPN and 24% of PDAC. Marked upstream dilatation of the main pancreatic duct in ITPN with an invasive carcinoma component was also reported by Kim et al.^28^. The authors suggested that marked pancreatic duct dilatation in ITPN is indicative of an invasive carcinoma, which could be due to impacted pancreatoliths associated with a very slow-growing intraductal tumor. However, there were no intraductal pancreatoliths in our patient sample.

In the same study by Motogusi et al.^30^, a 2-tone duct sign, which refers to intraductal solid tumor growth, was reported to be a finding suggestive for ITPN. They found the 2-tone duct sign to be present in 70% of patients on CT, in 63% of patients on MRI, and 100% of patients on endoscopic ultrasound (EUS). We were able to verify this imaging finding in 3 out of 12 patients (25%). However, a limitation of our study was that EUS images were not available for analysis. Similarly, Kim et al.^28^ reported that EUS can detect an intaductal solid component in all lesions, suggesting that EUS is more sensitive than CT or MRCP in this regard. Likewise, Efthymiou et al.^31^ stated that the high resolution of EUS and close proximity to the target lesion of the pancreas could explain the higher sensitivity for detection of small solid tumors in the duct.

In our case series, the majority of ITPNs (83%) were visualized as a solid tumor with moderate hypo- or isoenhancement, and the CT attenuation values of ITPN were significantly higher compared to PDAC. Interestingly, some authors also reported overlapping imaging features of ITPN with chronic autoimmune pancreatitis, PDAC, and IPMN^28,30^. Kim^28^ found that ITPN was mimicking chronic autoimmune pancreatitis or pancreatic ductal adenocarcinoma in 3 patients (37.5%) on multimodality imaging. In our study, the preoperative imaging diagnosis included mucinous cystic neoplasm (n = 1), a solid intrapancreatic lesion (n = 5), pancreatic ductal adenocarcinoma (n = 3), isoattenuating pancreatic ductal adenocarcinoma (n = 2), and IPMN of the mixed-duct type (n = 1). In no patient, ITPN was suspected on preoperative imaging.

Our study shows that the lesions’ margins and the CT density in the arterial and venous phase are the key imaging features for differentiation of ITPN with associated invasive carcinoma and conventional PDAC. We found that ITPN are more often well-circumscribed and have significantly higher HU values compared to PDAC (mean HU values of ITPN 68 ± 15.6 HU and mean HU values of PDAC 40.1 ± 13.6 HU in the venous phase). Also, a significant negative weak correlation was found between the CT density of the lesion and the degree of duct dilatation. It could be explained by lower density within more aggressive and invasive tumors, which have more prominent duct infiltration. Further studies on a larger number of patients are needed to verify our hypothesis.

To date, no clinical trials are available on treatment of ITPN^17^. Most authors consider oncological resection the treatment of choice for patients with ITPN with associated invasive carcinoma^17^. Since ITPN with associated invasive carcinoma seems to be less aggressive than conventional PDAC, less extensive surgical approaches with potentially lower surgery-related morbidity could be feasible. Therefore, accurate preoperative imaging diagnosis could be beneficial in the future.

There are several limitations to our study. The major limitation is the lack of standardization of CT and MRI protocols due to the retrospective design of our study. Another limitation is the relatively small sample size of ITPN lesions, which is, however, comparable to previous studies on ITPN and can be explained by the rarity of the disease. Multicenter studies involving a larger number of patients are needed to overcome this limitation. Finally, we didn’t compare ITPN of the pancreas with IPMN, since all ITPN from our study presented with an associated invasive carcinoma component and more often appeared as solid lesions, which could be misdiagnosed as classic PDAC.

## Conclusion

In the present study, we identified imaging features of ITPN with associated invasive carcinoma, which are rare tumors of the pancreas and lacking reliable imaging features in the existing literature. We found that ITPN lesions with invasive carcinoma appear as comparatively well-circumscribed solid lesions with significantly higher CT attenuation values on the enhanced images compared to conventional PDAC. This renders isodense PDAC the main differential diagnosis. The results from our study imply that the presence of an isodense or moderately hypodense lesion with well-circumscribed margin favors the diagnosis of ITPN with invasive carcinoma over conventional PDAC. Considering the rarity of ITPN, correct preoperative diagnosis of ITPN will remain challenging. Being familiar with the CT and MRI features of these rare pancreatic tumors is essential for radiologists to improve preoperative imaging diagnosis and narrow the differentials. Future studies with larger sample sizes are needed to further define discriminating imaging features of ITPN with associated invasive carcinoma versus conventional PDAC.

## Materials and methods

### Ethics approval and contest

Our study was performed according to the Declaration of Helsinki. The study protocol was approved by the ethics committee of the medical faculty of Heidelberg University (S-011/2015). Due to its retrospective design, the need of informed consent for the patients was waived by the Review board of Heidelberg University.

### Patient characteristics

The database of the Clinic for Diagnostic and Interventional Radiology was searched retrospectively for patients who had undergone surgical resection of ITPN between March 2003 and March 2018 and who had available imaging examinations before surgery. Clinical data and histopathological reports of these patients were retrieved from the hospital information system (ISH Med/SAP, Walldorf, Germany).

Twelve patients (7 male patients and 5 female patients) with histologically verified ITPN were included in the study. The final histopathological diagnosis was ITPN in all patients, all of whom had an associated invasive carcinoma. In one patient, the initial histopathological report had favored acinar cell carcinoma, but the diagnosis of ITPN was established by a reference pathologist.

We defined a control group of 43 patients with histologically verified pancreatic ductal adenocarcinoma, selected from our hospital database as matched pairs (matched for patient age and sex), who had undergone surgical resection in our institution from November 2016 to April 2019 without previous neoadjuvant therapy. We didn’t match for tumor stage since we aimed to compare imaging features of the average ITPN-carcinoma-patient versus the average conventional-PDAC-patient at initial diagnosis and ITPN with invasive carcinoma are often diagnosed at an earlier stage than most conventional PDACs.

### CT and MRI scans

Preoperative imaging studies were available for all patients. Imaging analysis was performed independently from routine radiological reports. Due to the retrospective nature of our study, the scanning protocols of CT and MRI were not standardized. CT examinations were performed with either 16 or 64 row scanners. CT scans were available for 10 patients; all of them included non-contrast images and two-phase contrast-enhanced images (arterial and portal venous phase) after intravenous injection of nonionic iodinated contrast medium. Arterial phase was defined by full enhancement of hepatic arteries as well as absence of antegrade enhancement of hepatic vein and portal venous phase was defined by full enhancement of portal veins and antegrade enhancement of hepatic veins. Slice thickness was 3 and 5 mm with reconstruction intervals of 3 and 5 mm for 16 and 64 row scanners, respectively. Effective tube current was 150–250 mAs with 120 kVp. All images were reconstructed using a soft tissue convolution Kernel. Coronal and sagittal reconstructions were available solely for venous phase images in 10 patients and for both arterial and venous phases in 4 patients.

MRI with magnetic resonance cholangiopancreatography (MRCP) was available for 8 patients and was performed on a 1.5 T scanner using a body coil. All MRI studies included breath-hold axial T1 weighted images (T1WI), axial T2 weighted images (T2WI) without fat suppression, and coronal T2WI with or without fat suppression (FS). MRCP images were obtained with a two-dimensional (2D) or three-dimensional (3D) technique. T1WI after contrast injection were obtained with dynamic protocol after an injection of extracellular contrast agent as a bolus injection of 0.2 ml/kg gadolinium chelate. Diffusion-weighted images (DWI) were available for 6 patients and were performed with b-values of 0, 50, and 800 s/mm^2^.

In 6 patients both preoperative CT and MRI studies were available. Mean time interval between the latest imaging and surgery was 31 days (range 1–220 days). Excluding one patient with the longest interval of 196 days, the mean interval was 14 days (range 1–36 days).

### Image analysis

All images were reviewed by two board-certified radiologists (EK and MK) with 7 and 20 years of experience in abdominal and oncologic imaging using institutional PACS (GE Healthcare, Chicago, IL, USA). Both radiologists were blinded to the results of histological examinations. Afterwards, discrepancies in image interpretation were resolved by consensus between the two radiologists.

The following imaging features were analyzed: location (head, body, tail, head/body, or body/tail), size (cm), boundaries of the lesion (well-circumscribed/unsharp), presence of a pseudocapsule, mural nodule, wall thickening, HU-values on native and contrast-enhanced (CE) images, signal intensities on T2WI, T2WI with FS and T1WI, enhancement in comparison to adjacent non-neoplastic pancreatic parenchyma, diameter of adjacent main pancreatic duct (mm), duct abruption, parenchymal atrophy, pancreatolithiasis, restricted diffusion in MRI, lymphadenopathy, presence of metastasis and biliary obstruction.

### Statistical analysis

Data were recorded using a spreadsheet program (Microsoft Office Excel 2019, Microsoft Corporation, Redmond, WA, USA) and a descriptive analysis was applied.

Statistical data analysis was performed using Medcalc V.20.110 for Windows (Medcalc Software, Ostend, Belgium) and SPSS V.23 for Mac (SPSS, Chicago, Illinois, USA). The data are presented as mean values with standard deviation (SD). The Mann–Whitney U-test was performed to compare continuous variables between ITPN and PDAC cases. The chi-squared test was used to compare categorical variables between ITPN and PDAC cases. A multiple logistic regression analysis was performed to predict the lesion entity based on their imaging characteristics. Progression-free survival was determined based on follow-up CT scans. Differences in progression-free survival were compared using the log-rank test.

The significance level for statistical testing was set at p < 0.05.

## Supplementary Information

Supplementary Legends.

Supplementary Figure 1.
