# Supplementary material for: Imaging features of intraductal tubulopapillary neoplasm of the pancreas and its differentiation from conventional pancreatic ductal adenocarcinoma
Source: Sci Rep. 2022 Sep 16;12:15557. doi: 10.1038/s41598-022-19517-6 (PMC9481632; doi:10.1038/s41598-022-19517-6)
Supplement: Supplementary file 1 — Supplementary Legends. [file 41598_2022_19517_MOESM1_ESM.docx]

**Supplementary Figure 1. Kaplan-Meier curves for progression-free survival of patients with ITPN with associated invasive carcinoma and patients with conventional PDAC.**

Two out of four ITPN with associated carcinoma patients with available follow-up data showed progression 130 days (distant metastases) and 1478 days (local recurrence) after surgery. Follow-up data regarding progression-free survival were available in twenty-four patients with conventional PDAC. Out of these twenty-four patients, progression was detected in seventeen patients resulting in a median progression-free survival of 292 days (95% confidence interval: 149 to 618 days) after surgery (local recurrence in seven patients, distant metastases in seven patients, both in three patients). According to the log-rank test, progression-free survival was non-significantly longer in patients with ITPN with associated carcinoma than in patients with conventional PDAC (p = 0.1969).
