# Supplementary figures and images for: Imaging features of intraductal tubulopapillary neoplasm of the pancreas and its differentiation from conventional pancreatic ductal adenocarcinoma
Source: Sci Rep. 2022 Sep 16;12:15557. doi: 10.1038/s41598-022-19517-6 (PMC9481632; doi:10.1038/s41598-022-19517-6)

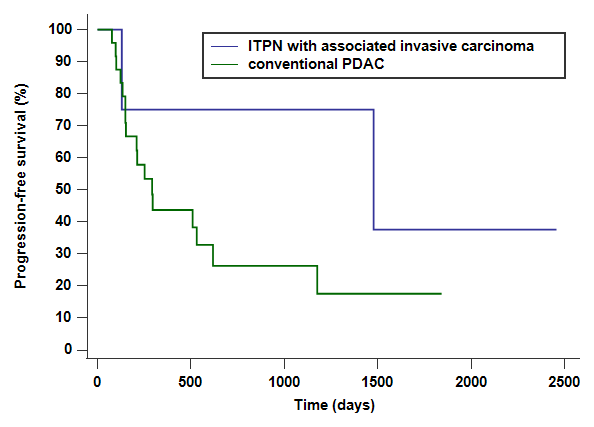

Supplement: Supplementary file 2 — Supplementary Figure 1. [file 41598_2022_19517_MOESM2_ESM.tif]
